# Pregnancy Outcome of Multiparous Women Aged over 40 Years

**DOI:** 10.1155/2013/287519

**Published:** 2012-12-29

**Authors:** Seda Ates, Gonca Batmaz, Osman Sevket, Taner Molla, Cem Dane, Banu Dane

**Affiliations:** ^1^Department of Obstetrics and Gynecology, Bezmialem Vakif University, 34093 Istanbul, Turkey; ^2^Bezmialem Vakif University, Adnan Menderes Bul. Vatan Cad., 34093 Istanbul, Turkey; ^3^Department of Obstetrics and Gynecology, Haseki Education and Research Hospital, Istanbul, Turkey

## Abstract

*Objective*. The aim of this study was to evaluate the effect of maternal age on prenatal and obstetric outcome in multiparaous women. *Materials and Methods*. A retrospective case control study was conducted, including women aged 40 years and over (study group, *n* = 97) who delivered at 20 week's gestation or beyond and women aged 20–29 years (control group, *n* = 97). *Results*. The mean age of women in the study group was 41.2 ± 1.7 years versus 25.4 ± 2.3 years in the control group. Advanced maternal age was associated with a significantly higher rate of hypertension, diabetes mellitus, fetal complication, and 5-minute Apgar scores <7 (*P* < 0.05). Caeserean section rate, incidence of placental abruption, preterm delivery, and neonatal intensive care unit admission were more common in the older group, but the differences were not statistically significant. *Conclusions*. Advanced maternal age is related to maternal and neonatal complications.

## 1. Introduction

Many women increasingly delay pregnancy and childbirth into their fourth decade of life because of different reasons, such as delay in marriage, educational and professional reasons [1]. Some of them experience pregnancy unwillingly because of inappropriate use of contraceptive methods [2].

Advanced maternal age has been regarded as a risk factor for complications in pregnancy. The association between advanced maternal age and increased risk of chromosomal abnormalities and spontaneous abortion has been well documented in studies [3, 4].

There are different publications in the literature on pregnancy outcomes of women aged 40 years or older. Some authors have reported that advanced maternal age has been associated with preterm delivery, low birth weight, perinatal mortality, and higher frequency of cesarean section [5, 6]. But others have reported no obvious difference in the perinatal outcomes [7], obstetric outcomes [8], birth weight, Apgar score, and admission to neonatal intensive care unit [9] between younger and older mothers. A systematic review stated that advanced aged mothers have an increased risk of stillbirth. The mechanism of the increase in stillbirth risk with advanced maternal age is uncertain [10].

The aim of this study was to determine the effect of maternal age on obstetric and perinatal outcome in multiparous women aged at least 40 year old with multiparous women aged 20–29 years.

## 2. Materials and Methods

This is a retrospective study for women delivered at gestational ages of >20 weeks in Bezmialem Vakif University and Haseki Education and Research Hospital between October 2010 and December 2011. We reviewed the obstetric records of 97 multiparous women aged 40 or above at the time of delivery and compared with a control group which consisted of consecutive 97 multiparous women with ages 20–29 years who delivered at the same period of time. The following data, including maternal age at the time of delivery, gravidity, parity, gestastional age, antenatal complications (pregnancy-induced hypertension, diabetes, preterm delivery, delivery <24 weeks, abruptio placenta, small for gestational age, large for gestational age), mode of delivery, indications for ceaserean sections, intrapartum, and neonatal outcome (fetal distress, fetal complication, fetal malformation, birth weight, Apgar score, neonatal intensive care unit admission, and stillbirth) were collected and compared with a control group.

Maternal age was considered as the age at the time of delivery. Gestational age was determined on the basis of either date of last menstrual period or ultrasound examination. Diastolic blood pressure >90 mmHg was defined as hypertension in pregnancy (essential or pregnancy-induced hypertension). Diabetes mellitus was considered as history of diabetes (based on medical records) or gestational diabetes. Like other studies, we combined chronic hypertension, pregnancy-induced hypertension, and eclampsia into one condition called HDP (hypertensive disorders of pregnancy), and we combined gestational and established diabetes into another [11].

Abruptio placentae refers to the premature separation of the normally implanted placenta from the uterus. Preterm birth was defined delivery before 37 completed gestastional weeks. Small for gestational age (SGA) was defined as <10th percentile of birth weight for gestational age and large for gestational age (LGA) as ≥90th percentile. Stillbirth was defined as intrauterine death of a fetus weighing at least 500 g after 20 completed weeks of gestation. Low Apgar score was defined as a score of less than seven at 5 min following birth. Fetal complications include stillbirth, neonatal intensive care unit admission, and fetal malformation.

Statistical analysis was performed using the MedCalc for Windows, version 8.1.00 (MedCalc Software, Mariakerke, Belgium). Data were presented as means ± standard deviations or numbers of subjects and percent. Chi-square and Fisher's exact test were used to compare the variables. Odds ratio (OR) and 95% confidence intervals (CI) are presented in order to analyse the risk related with advanced maternal age, and *P* < 0.05 was regarded as statistically significant.

## 3. Results

We reviewed 97 records of women aged 40 years old or more. The mean age of women in the study group was 41.2 ± 1.7 years and that in the control group was 25.4 ± 2.3 years at the time of delivery. The older mothers had a higher mean gravidity compared with for the control group (4.9 ± 2.5 versus 2.6 ± 1, *P* < 0.0001). Gestational age at the delivery was lower among the older mothers compared with the younger women (Table 1).

The incidence of hypertension and diabetes mellitus was significantly higher in older women compared with the younger group. Abruptio placenta, delivery before 24 weeks, preterm delivery, and LGA were seen more often among older mothers but the difference did not reach the level of significance (Table 2).

The infants of the older mothers showed a higher incidence of stillbirth (5.1% versus 0%), admission to the neonatal intensive care unit (5.1% versus 1.03%), and fetal malformation (3.09% versus 0.8%) than younger mothers, but the differences were not statistically significant. The results showed that the rate of fetal complication (*P* = 0.021), 5-minute Apgar scores <7 (*P* = 0.032) were significantly higher among older mothers (Table 3).

The cesarean section delivery rate was 65.9% in the study group and 55.6% in the control group, respectively. The repeat cesarean sections in both groups were performed after the onset of labour, no programmed section was performed in any of the groups. The major indication for cesarean delivery in this study included previous cesarean section in both groups.

The incidence of cesarean section for fetal distress (*P* = 0.017) and fetal macrosomia (*P* = 0.041) was significantly higher in the group of older mothers than in the younger mothers (Table 4).

The risk of hypertension (OR, 6.7; 95% CI, 1.45–30.8; *P* = 0.014), diabetes mellitus (OR, 18.5; 95% CI, 1.05-325; *P* = 0.046), and 5 minute Apgar score <7 (OR, 8.62; 95% CI, 1.05–70.3; *P* = 0.044) were higher among the older group. The rate of cesarean delivery was higher in older group (OR, 1.54; 95% CI, 0.86–2.7; *P* = 0.14) but the difference was not statistically significant (Table 5).

## 4. Discussion

Our study confirms a significant higher incidence of hypertension and diabetes mellitus among pregnant women age 40 and older, which has been reported in other studies [12–14]. The prevalence of diabetes and hypertension are increased by age and considered to induce vascular endothelial damage that occurs with aging [15].

Contrary to the literature [12, 16, 17], the cesarean section was slightly higher in the older multiparous mothers aged 40 and older compared with younger multiparous mothers in our study. Many reports have described a higher incidence of cesarean delivery among the nulliparous women age 40 or older [5, 18]. Elderly primiparous women frequently have a long history of infertility and the probability of this being the only pregnancy may influence a physician's decisions to perform caesarean delivery. This suggested that parity imposed a more important effect on the incidence of cesarean section than maternal age. In this study, the incidence of placental abruption was higher in women aged 40 years or older than younger women, this difference was not significant.

The primary indication for cesarean section in this study was previous caesarean delivery in both groups. The main reason for the high caesarean section rates in the control group (90.7%) is related to the previous caesarean deliveries. Caesarean section rates are increasing recently in many countries [19]. The caesarean section rates in these young women who had previous caesarean sections is high since the indications of caesarean section is also increased in our country. The women in the study group are older and thus they may not be as affected as the younger control group from this rise in the changing trend of the delivery route. Additionally, the high rate of cesarean delivery in both groups is due to the fact that our clinic is a tertiary center. Fetal distress constituted 20 and 3.7% of the indications for caesarean section in older and younger mothers, respectively. The rate of cesarean section for fetal macrosomia is accounted for 12.5% in the study group and 1.03% in the control group. This significantly higher rate of cesarean section may be related with diabetes mellitus which is clearly regarded as a cause of macrosomia [20].

Mean gestational age for the older group at delivery was significantly lower than that for the younger group. This fact may be associated with maternal or fetal problems such as diabetes, chronic hypertension, and fetal distress [18] which is more frequently seen in older mothers.

The rate of stillbirth was higher among the older group although the difference was not significant. Stillbirth occured in 5 cases in the study group in which down syndrome was the reason of death in one case. The rest of the patients did not accept the autopsy. The risks of aneuploidy and fatal congenital anomalies increase with maternal age and, despite antenatal screening, they are likely to have contributed to the increased rate of stillbirth [21]. The failure of uterine vasculature to adapt to the increased hemodynamic demands of pregnancy has also been suggested as a cause of fetal death in women aged 40 years and older [20]. This study shows that a 5-minute Apgar score <7, which is a better indicator of long-term neonatal outcome, was higher in the older mothers compared with the younger ones. Additionally the rates of stillbirth and fetal complication was higher in the older group.

Neonatal intensive care unit admission was more frequent among in the older women although the difference was not statistically significant (*P* = 0.21). It may be explained by the increased incidence of fetal distress, with lower Apgar scores and fetal distress among older patients.

Advance maternal age was also a risk factor for preterm delivery in our study. The rates of preterm delivery, delivery <24 week were more frequently seen among in the older mothers, although the difference was not statistically significant. Maternal and fetal complications such as hypertensive diseases, diabetes, and fetal distress may contribute to the increased risk of preterm delivery among the multiparous women aged 40 years and older [14].

Mean birth weight was almost the same for the two age groups. Therefore, this would suggest that maternal age may not affect birth weight as much as other factors [4].

In conclusion, pregnancy in older multiparous women seem to have higher rates of obstetric complications and adverse birth outcomes, such as hypertension, diabetes, and lower Apgar scores. Women should be informed that the risk of pregnancy complications and adverse birth outcome increases with age. Additional studies are needed to examine the relationship between maternal age and maternal and fetal outcomes and the mechanisms on how advanced maternal age increases the risk of adverse birth outcomes in different subgroups of women.

## Figures and Tables

**Table 1:** Patients characteristics.

	Study group (*n*: 97)	Control group (*n*: 97)	*P* value
Age (years)∗	41.2 ± 1.7	25.4 ± 2.3	<0.0001
Gravidity∗	4.9 ± 2.5	2.6 ± 1	<0.0001
Gestational age (weeks)∗	37.8 ± 3.2	38.6 ± 1.7	0.03

^*^Value are mean ± SD.

**Table 2:** Antenatal complications.

	Study group (*n*: 97)	Control group (*n*: 97)	*P* value
Hypertension	12 (12.3)	2 (2.06)	0.01
Diabetes mellitus	8 (8.2)	0 (0)	0.01
Abruptio placenta	3 (3.09)	0 (0)	0.24
Delivery <24 weeks	2 (2.06)	0 (0)	0.47
Preterm delivery	15 (15.4)	10 (10.3)	0.39
SGA	7 (7.2)	11 (11.3)	0.46
LGA	13 (13.4)	9 (9.27)	0.49

PIH: Pregnancy-induced hypertension, SGA: Small for gestational age, LGA: Large for gestational age.

Data are presented *n* (%) Chi-square, Fisher's exact test.

**Table 3:** Fetal and neonatal outcome.

	Study group (*n*: 97)	Control group (*n*: 97)	*P* value
Stillbirth	5 (5.1)	0 (0)	0.07
NICU	5 (5.1)	1 (1.03)	0.21
Fetal malformation	3 (3.09)	1 (0.8)	0.52
Fetal complication	12 (12.3)	2 (2.6)	0.021
Fetal weight (gram)∗	3190 ± 819	3264 ± 487	0.44
5-minute Apgar score <7	8 (8.2)	1 (0.8)	0.032

^*^Value are mean ± SD.

Data are presented *n* (%), NICU: neonatal intensive care unit (1 Down S., 1 mega cysterna manga, 1 NTD, 1 fetal ascites.

**Table 4:** Cesarean section indications.

Indication	Study group (*n* : 64)	Control group (*n* : 54)	*P* value
Previous cesarean section	32 (50)	49 (90.7)	<0.0001
Fetal distress	13 (20)	2 (3.7)	0.017
Peeclampsia	6 (9.3)	2 (3.7)	0.4
Malpresentation	2 (3.1)	1 (1.03)	0.89
Fetal macrosomy	8 (12.5)	1 (1.03)	0.041
Failure to progress	2 (3.1)	2 (3.7)	0.74
Previous myomectomy	1 (1.5)	0 (0)	0.9
Cord prolapse	1 (1.5)	0 (0)	0.9
Fetal anomaly	1 (1.5)	0 (0)	0.9

Data are presented *n* (%). Some of the cases had more than 1 indication.

**Table 5:** Comparison of maternal and neonatal complications between women aged >40 years and those aged 20–29 years.

	Study group (*n*: 97)	Control group (*n*: 97)	OR (95% CI)	*P* value
Hypertension	12 (12.3)	2 (2.06)	6.7 (1.45–30.8)	0.014
Diabetes mellitus	8 (8.2)	0 (0)	18.5 (1.05–325)	0.046
Preterm delivery	15 (15.4)	10 (10.3)	1.59 (0.67–3.7)	0.28
Cesarean delivery	64 (65.9)	54 (55.6)	1.54 (0.86–2.7)	0.14
SGA	7 (7.2)	11 (11.3)	0.60 (0.22–1.64)	0.32
LGA	13 (13.4)	9 (9.27)	1.51 (0.61–3.7)	0.36
5 minute Apgar score <7	8 (8.2)	1 (0.8)	8.62 (1.05–70.3)	0.044
Intrauterinefetal death	5 (5.1)	0 (0)	11.5 (0.63–212.6)	0.09

^*^Data are presented *n* (%), OR: odds ratio, CI: confidence interval.

## References

[1] Balasch J., Gratacós E. (2011). Delayed childbearing: effects on fertility and the outcome of pregnancy. *Fetal Diagnosis and Therapy*.

[2] Obed S. A., Armah J. O., Wilson J. B. (1995). Advanced maternal age and pregnancy. *West African Journal of Medicine*.

[3] Bayrampour H., Heaman M., Duncan K. A., Tough S. (2012). Advanced maternal age and risk perception: a qualitative study. *BMC Pregnancy Childbirth*.

[4] Ziadeh S., Yahaya A. (2001). Pregnancy outcome at age 40 and older. *Archives of Gynecology and Obstetrics*.

[5] Ludford I., Scheil W., Tucker G., Grivell R. (2012). Pregnancy outcomes for nulliparous women of advanced maternal age in South Australia, 1998–2008. *Australian and New Zealand Journal of Obstetrics and Gynaecology*.

[6] Koo Y. J., Ryu H. M., Yang J. H. (2012). Pregnancy outcomes according to increasing maternal age. *Taiwanese Journal of Obstetrics and Gynecology*.

[7] Diejomaoh M. F. E., Al-Shamali I. A., Al-Kandari F., Al-Qenae M., Mohd A. T. (2006). The reproductive performance of women at 40 years and over. *European Journal of Obstetrics Gynecology and Reproductive Biology*.

[8] Smit Y., Scherjon S. A., Treffers P. E. (1997). Elderly nulliparae in midwifery care in Amsterdam. *Midwifery*.

[9] Takahashi H., Watanabe N., Sugibayashi R. (2012). Increased rate of cesarean section in primiparous women aged 40 years or more: a single-center study in Japan. *Archives of Gynecology and Obstetrics*.

[10] Huang L., Sauve R., Birkett N., Fergusson D., van Walraven C. (2008). Maternal age and risk of stillbirth: a systematic review. *Canadian Medical Association Journal*.

[11] Delbaere I., Verstraelen H., Goetgeluk S., Martens G., de Backer G., Temmerman M. (2007). Pregnancy outcome in primiparae of advanced maternal age. *European Journal of Obstetrics Gynecology and Reproductive Biology*.

[12] Franz M. B., Husslein P. W. (2010). Obstetrical management of the older gravida. *Women's Health*.

[13] Tabcharoen C., Pinjaroen S., Suwanrath C., Krisanapan O. (2009). Pregnancy outcome after age 40 and risk of low birth weight. *Journal of Obstetrics and Gynaecology*.

[14] Jahromi B. N., Husseini Z. (2008). Pregnancy outcome at maternal age 40 and older. *Taiwanese Journal of Obstetrics and Gynecology*.

[15] Eisenberg V. H., Schenker J. G. (1997). Pregnancy in the older woman: scientific and ethical aspects. *International Journal of Gynecology and Obstetrics*.

[16] Han A. R., Kim H. O., Cha S. W. (2011). Adverse pregnancy outcomes with assisted reproductive technology in non-obese women with polycystic ovary syndrome: a case-control study. *Clinical and Experimental Reproductive Medicine*.

[17] Tomić V., Grizelj B., Zadro M. (2008). Perinatal outcome in primiparous women aged 35 and older: a case-control study. *Medicinski Arhiv*.

[18] Gilbert W. M., Nesbitt T. S., Danielsen B. (1999). Childbearing beyond age 40: pregnancy outcome in 24,032 cases. *Obstetrics and Gynecology*.

[19] Katikireddi S. V., Gorman D. R., Leyland A. H. A comparison of trends in caesarean section rates in former communist (transition) countries and other European countries.

[20] Evers I., de Valk H., Mol B., Ter Braak E., Visser G. (2002). Macrosomia despite good glycaemic control in type I diabetic pregnancy; results of a nationwide study in The Netherlands. *Diabetologia*.

[21] Jolly M., Sebire N., Harris J., Robinson S., Regan L. (2000). The risks associated with pregnancy in women aged 35 years or older. *Human Reproduction*.

